# Oncolytic Viruses as Therapeutic Tools for Pediatric Brain Tumors

**DOI:** 10.3390/cancers10070226

**Published:** 2018-07-09

**Authors:** Maider Varela-Guruceaga, Sonia Tejada-Solís, Marc García-Moure, Juan Fueyo, Candelaria Gomez-Manzano, Ana Patiño-García, Marta M. Alonso

**Affiliations:** 1 The Health Research Institute of Navarra (IDISNA), 31008 Pamplona, Spain; mvarelag@unav.es (M.V.-G.); stejada@unav.es (S.T.-S.); mgmoure@unav.es (M.G.-M.); apatigar@unav.es (A.P.-G.); 2 Department of Pediatrics, University Hospital of Navarra, 31008 Pamplona, Spain; 3 Department of Neurosurgery, University Hospital of Navarra, 31008 Pamplona, Spain; 4 Department of Neuro-Oncology, The University of Texas MD Anderson Cancer Center, Houston, TX 77030, USA; jfueyo@mdanderson.org (J.F.); cmanzano@mdanderson.org (C.G.-M.); 5 Department of Genetics, The University of Texas MD Anderson Cancer Center, Houston, TX 77030, USA

**Keywords:** oncolytic virus, pediatric brain tumors, immunostimulation

## Abstract

In recent years, we have seen an important progress in our comprehension of the molecular basis of pediatric brain tumors (PBTs). However, they still represent the main cause of death by disease in children. Due to the poor prognosis of some types of PBTs and the long-term adverse effects associated with the traditional treatments, oncolytic viruses (OVs) have emerged as an interesting therapeutic option since they displayed safety and high tolerability in pre-clinical and clinical levels. In this review, we summarize the OVs evaluated in different types of PBTs, mostly in pre-clinical studies, and we discuss the possible future direction of research in this field. In this sense, one important aspect of OVs antitumoral effect is the stimulation of an immune response against the tumor which is necessary for a complete response in preclinical immunocompetent models and in the clinic. The role of the immune system in the response of OVs needs to be evaluated in PBTs and represents an experimental challenge due to the limited immunocompetent models of these diseases available for pre-clinical research.

## 1. Introduction

### 1.1. Pediatric Brain Tumors

Cancer is the leading cause of death by disease in children (0–18 years of age), and central nervous system (CNS) tumors are mainly associated with these deaths in this age group [1,2,3]. The most common brain tumor in children is glioma (52.9%) followed by embrional tumors (15%) [4]. The prognosis of many pediatric tumors has improved in recent years, however, around 65% of children with atypical teratoid/rhabdoid tumors (AT/RT) or high risk medulloblastoma and more than 95% of children with brainstem glioma succumb to their disease [5].

The standard treatment of pediatric brain tumor (PBTs) is surgery, to remove the tumor, followed by chemotherapy and radiotherapy. However, the resection is not always easy due to the localization and characteristics of the tumor. Moreover, treatment with chemotherapeutic agents and radiation therapy not only affect cancer cells but also normal cells, producing high toxicity and, sometimes, severe sequelae after including neurological, endocrine, and neurosensory impairments [6]. Furthermore, many patients develop chemoresistance which significantly reduce their survival. For these reasons, there is an urgent need for new therapies that could decrease the toxicity and improve the survival rates in PBTs.

### 1.2. Oncolytic Viruses

Oncolytic viruses (OVs) are promising therapeutic tools for solid tumors due to their many biological advantages compared to traditional approaches including: (1) the selective replication of the OVs in cancer cells without affecting normal cells; (2) the lack of resistance mechanism by the targeted cells and (3) the capacity of the OV to spread throughout the tumor once a few cells have been infected (4) the capacity to trigger an immune response against the tumors [7,8,9,10]. OVs represent a potential therapy for several tumors, including PBTs, due to their capacity to target cancer stem cells (CSC) which are, in theory, the cells responsible for tumor growth [11]. In this sense, Friedman et al. showed that glioma CD133(+) cells, enriched in CSC, were as sensitive as glioma cells CD133(−) to different strains of oHSV including G207 and M002 [12]. Additionally, the oncolytic adenovirus, CRAd-Survivin-pk7, has the ability to efficiently replicate and induce cytotoxicity in a panel of passaged and primary CD133(+) adult glioma stem cells [13]. Other types of OVs including Seneca Valley Virus and myxoma virus have also shown the ability to target CSC (see reference [11] for review).

Several types of viruses have been studied as a possible treatment for brain tumors in childhood. Some of these viruses have natural tropism for tumor cells, and others have been attenuated to improve the safety or they have been genetically modified to render them tumor-specific. In this regard, myxoma and Newcastle disease viruses (NDV) are examples of viruses which naturally replicate in most cancer cells [9,10]. On the other hand, measles and vaccinia viruses have been attenuated to avoid the replication in normal tissues and, therefore, increase the safety of the therapy [10,14]. Finally, adenoviruses (Ads) and herpes simplex virus (HSV) have been widely tested in preclinical and clinical models of different pediatric cancers and are examples of viruses that have been genetically modified to take advantage of the aberrant activation of signaling pathways in cancer cells to enter and/or replicate while sparing normal cells [7,15].

**Figure 1:**
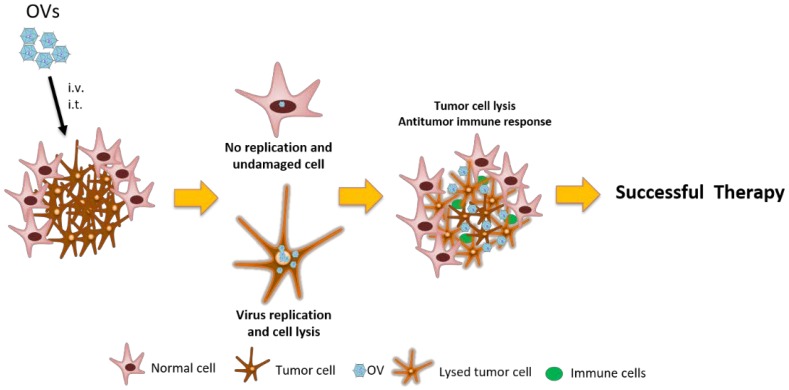
Mechanism of antitumoral effect of oncolytic viruses (OVs). The administration of OVs could be intravenous or intratumoral. Once the virus reaches the tumor is able to infect both normal and tumor cells but only replicates and lyses the tumor cells. Besides the potent cytolytic effect of the virus, the generation of an antitumor immune response is crucial to the complete eradication of the tumor.

The antitumor response of OV is mediated by two main mechanisms: (1) the direct lysis of cancer cells due to the virus replication and, (2) the increase or activation of an immune response against the tumor as a result of the immunogenic cell death induced by the virus and the release of neoantigens to the microenvironment [8]. However, the antitumor immunity should be more important than the clearance of the virus by the host immune response in order to be efficient and produce the shrinkage of the tumor (Figure 1) [15].

The use of viruses as therapeutic alternatives for brain tumors, has been investigated since the early 1990s, when Martuza and colleagues published a mutated HSV with a deletion of the enzyme thymidine kinase gene [16]. The re-engineered HSV showed a potent lytic effect over two human glioblastoma cell lines and prolonged the survival of nude mice bearing the U87 glioma cell line orthotopically [16]. This work opened the field to numerous studies using different OVs as possible treatments for PBTs. In this review, we will summarize the different viruses that have been tested in different types of PBTs (Table 1) and the clinical relevance of these approaches.

## 2. Oncolytic Viruses for PBTs

### 2.1. Medulloblastoma

Medulloblastoma (MB) is an embryonal brain tumor located in the cerebellum that affects children between 6 and 11 years of age [38,39]. MB is the most frequent brain tumor in childhood representing 16% of all PBTs [39]. Molecular studies have determined that MB is not a single disease and has been classified into four subgroups, termed Wingless (Wnt), Sonic Hedgehog (SHH), Group 3, and Group 4 [40]. Tumors in the subgroup 3 and 4 have the highest rate of metastasis and the worst prognosis [19,41]. The standard treatment for MB is the surgical resection of the tumor followed by radiotherapy or/and chemotherapy which results in five-year survival rates of about 50% [39]. However, recovered patients often present long-term consequences due to the treatments, including neurological, endocrine, and neurosensory impairment underscoring the urgent need for novel and more specific therapies to improve the survival and the secondary effects of traditional treatments [19,41,42].

The use of OVs has showed satisfactory results in preclinical models of MB. Several types of viruses have been tested including HSV, parvovirus and measles virus (MV).

#### 2.1.1. Herpes Simplex Virus

Engineered oncolytic oHSV has been widely studied demonstrating clinical efficacy and safety in adult gliomas [19,43].

In 1996, the first study testing an OV for the treatment of a PBT, MB was published [17]. In this report, Lasner et al. constructed HSV-1 mutants with deletions in the *RL-1* gene that encodes the ICP34.5 protein which only allow the replication of the virus in proliferating cells without affecting normal cells. The neuroattenuated ICP34.5 mutant HSV-1 (variant-1716) significantly increased the survival of xenografted nude mice with the human MB cell line D283 [17]. Moreover, Friedman et al., recently evaluated two other oncolytic variants of the HSV-1, G207 and M002, in four human pediatric MB xenografts classified as Group 3 or 4, the most aggressive forms of the disease [19]. In vitro cytotoxicity assays showed that all xenografts tested were very sensitive to be killed by both oHSVs mutants, G207 and M002, with IC50s between 0.3 and 0.9 pfu/mL [19]. More importantly, mice bearing human MB exhibited a prolonged median survival time after treatment with a single administration of G207 or M002 [19]. Additionally, Studebaker et al. evaluated the oHSV variant, rRp450 which is deficient in the viral-encoded ribonucleotide reductase (ICP6) and expresses rat CYP2B1, an enzyme that activates the chemotherapeutic prodrug cyclophosphamide (CPA) [21]. In their work, orthotopic xenograft mouse models of MB treated with a single injection of rRp450 showed significantly longer survival than the control group [21]. Mice implanted with group 4 MB cells, D283 Med, showed 41.6% of complete responses at day 100 after tumor implantation followed by 27.3% of complete responses in Group 3 MB D425med- bearing mice [21].

All this evidence strongly supports the susceptibility of Groups 3 and 4 MB cells to oHSV. However, so far, the use of these viruses has not been evaluated in clinical trials for MB patients.

#### 2.1.2. Measles Virus

MV is a negative strand RNA virus that induces the formation of multinuclear cell aggregates known as syncytia, which result in cell death [44]. In contrast to the wild-type MV, vaccine strains are attenuated and have a good record of safety in other tumors [45,46,47]. Studebaker et al. evaluated the attenuated Edmonston’s strain of MV in five human cell lines of MBs [22]. They demonstrated the expression of the MV receptor, CD46, in all cell lines tested. Moreover, MV induces the formation of syncytia and therefore produces MB cell death in vitro [22]. Furthermore, in an orthotopic D283 Med mouse model the administration of MV increased the survival compared with those treated with inactivated virus [22]. A few years later, the same group established a mouse model of disseminated MB which is associated with poor prognosis [48]. In this work, mice bearing MB cells in their right lateral ventricle showed tumor growth following a disseminated pattern in their ventricles and in both intracranial and spinal sub-arachnoid spaces. Intraventricular administration of MV reduced tumors size and showed a significant increase in the overall survival compared to control mice [48].

Due to the high vascularization of MBs and the capacity of MV to incorporate foreign genes into the viral genome, Hutzen et al. constructed an MV encoding the human and mouse variants of the antiangiogenic endostatin/angiostatin fusion protein in order to improve the antitumor effect [49]. However, the mice inoculated with D283 Med cells and subsequently treated with recombinant MV-E:A showed no significant benefit when compared to mice treated with wild-type MV [49].

#### 2.1.3. Parvovirus H1

The rodent parvovirus H-1 (H-1PV) is a single-stranded DNA virus with natural oncolytic properties [50]. The infectivity and replication of H-1PV was demonstrated in six human MB cell lines [24]. The infection of all cell lines with H-1PV reduced the viability and produced a lytic effect in vitro [24]. Lacroix et al. also showed that the cytotoxic effect of H-1PV could be mediated by the downregulation of genes involved in early neurogenesis including *ZIC1*, *FOXG1B*, *MYC*, and *NFIA* which are also associated with poor prognosis of MBs [24]. However, so far there are no reports about the efficacy of H-1PV in animal models of MB.

#### 2.1.4. Reovirus

Reovirus is a double-stranded RNA virus commonly isolated from the respiratory/gastrointestinal tracts of humans. Reovirus has the ability to preferentially infect and replicate in tumor cells taking advantage of the Ras-signaling pathway, usually upregulated in cancer [51,52]. Yang et al. showed that infection with reovirus induced cytotoxicity (more than 90% of dead cells) in five MB cell lines [26]. Furthermore, nude mice bearing the MB cell line, Daoy, in the cerebellum followed by the intratumoral administration of a single dose of reovirus, dramatically prolonged their survival and led to long-term survivors without histological evidence of residual tumor [26].

#### 2.1.5. Myxoma Virus

Myxoma virus is a poxvirus only pathogenic to European rabbits. Despite its narrow host range in nature, it has been shown to infect and kill various classes of human tumor cells including renal, prostate, ovarian cancers and gliomas [53]. In this regard, Xue et al. demonstrated that nine MB cell lines were infected and killed by myxoma virus [27]. Moreover, in vivo, the intratumoral administration of myxoma virus prolonged survival of Daoy tumor-bearing mice and led to 60% being long-term survivors [27].

#### 2.1.6. Adenovirus

Adenovirus (Ad) is a non-enveloped double-stranded DNA virus with an icosahedral capsid composed of up to seven different structural proteins [54,55]. There are 52 serotypes of human Ad so far, but the most used as OVs are serotypes 2 (Ad2) and 5 (Ad5). Whereas wild-type Ad can infect and replicate in both dividing and nondividing cells, producing respiratory, ophthalmic, or gastrointestinal illnesses in humans, the deletions in *E1A* or *E1B* Ad genes result in attenuated mutants which can replicate specifically in cancer cells with few side effects [56,57]. Efficient viral infectivity is dependent on the binding of the fiber knob of the virus to the coxsackievirus adenovirus receptor (CAR) on the cell surface [58]. In this sense, surgical biopsies of different tumors showed that MB and neuroblastomas exhibited higher expression of CAR than gliomas and other brain tumors [59]. In line with this finding Dr. Juan Fueyo and colleagues showed that the oncolytic Ad5 Delta-24, which harbors a 24-base pair deletion in the Rb-binding region of the *E1A* gene providing conditional replication in tumor cells [28,60], was able to infect and replicate efficiently in Daoy and D283 Med cells. Moreover, Delta-24 induced a total cytopathic effect in Daoy and D283 Med cells after 6 and 8 days of infection, respectively [28]. Despite these positive results, in vivo studies evaluating the effect of oncolytic Ad in MB animal models have not been performed so far.

##### Seneca Valley Virus-001

Seneca Valley virus (SVV)-001, also known as NTX-010, is a single-stranded RNA virus that belongs to the family of Picornaviridae. SVV-001 was first described as an OV by Reddy et al. in a study that showed that 13 out of 23 cell lines derived from small-cell lung cancers and seven of eight solid pediatric cancers, including MB cell lines, were at least 10,000-fold more sensitive to the cytolytic activity of the virus than normal human cell lines [61,62]. One of the advantages of SVV-001 is its ability to pass the blood–brain barrier and target solid tumors allowing the intravenous administration and therefore, making it a promising candidate for the treatment of brain tumors [62]. Yu et el, determined in two orthotopic xenograft models of anaplastic MB that a single intravenous (i.v.) injection of SVV-001 led to widespread infection in the tumor, resulting in a significant increase in survival in both models [32]. Indeed, the experiment showed complete elimination of the tumor in eight of the ten long-term survivors [32].

### 2.2. Atypical Teratoid Rhabdoid Tumors

Atypical teratoid rhabdoid tumors (AT/RTs) are highly aggressive embryonal central nervous system (CNS) tumors primarily occurring in very young children (<3 years of age) [63,64]. Histologically, AT/RT is a heterogeneous tumor with a combination of rhabdoid cells and neuroectodermal, epithelial, and mesenchymal elements [65]. Since loss of INI1 (SMARCB1) protein expression is observe in 98% of AT/RTs, this is the genetic signature to confirm the diagnostic. So far, there is no consensus about the optimal therapeutic management of this tumor and the different schedules of traditional modalities have been used including maximal surgical resection, intensive chemotherapy and radiotherapy, which is frequently limited to the age of patients [63]. However, the prognosis of patients with AT/RT is poor with an overall survival of 6–18 months [1]. In this sense, OVs have been proposed as a potential therapy for AT/RT patients. 

#### 2.2.1. Measles Virus

MV has been tested in two human AT/RT cell lines, BT-12 and BT-16, showing the ability to replicate and to induce a potent cytopathic effect [23]. Treatment with MV resulted in a significant increase in the overall survival of AT/RT xenografts (localized and disseminated) in athymic nude mice [23].

The preclinical data obtained about the safety and efficacy of MV in MBs and AT/RTs supported the opening of a multi-center, Phase I study (NCT02962167) to determine the safety and preliminary efficacy of attenuated MV-NIS administered directly into the tumor bed (for locally recurrent MB or AT/RTs patients) or into the subarachnoid space (for disseminated recurrent MBs or AT/RTs patients) [66]. The clinical trial is now in the recruiting phase [66].

#### 2.2.2. Double-Deleted Vaccinia Virus

Vaccinia virus is a double-stranded and enveloped lytic DNA virus that has shown a good safety profile and efficacy against several adult tumors including, gliomas, liver cancer and mamarian carcinoma [14,67,68,69]. Vaccinia virus was originally generated as an expression vector for the development of veterinary vaccines and in clinical trials for some human infectious diseases [14,70]. In order to increase the replication selectivity in cancer cells a “double-deleted” version of the Western Reserve strain (double-deleted VV (vvDD)) in two genes, the thymidine kinase (TK) and the vaccinia growth factor was generated [71]. The replication of vvDD is therefore dependent on cellular TK, which is usually increased by cell-cycle abnormalities in cancer cells [72].

Xueqing et al. demonstrated (in vitro) that vvDD is able to infect and induce cytotoxicity in AT/RT cell lines, BT-12, BT-16 and KCCF1 [34]. Moreover, they showed that vvDD significantly inhibits the growth of intracranial AT/RT tumors in CD-1 nude mice which is accompanied by an increase in the overall survival of AT/RT-bearing animals treated with vvDD compared to control mice [73].

#### 2.2.3. Herpes Simplex Virus

The oHSV has also been tested in preclinical models of AT/RT. Studebaker et al. reported the therapeutic effect of the clinically available oHSV rRp450 in AT/RT cell lines BT-12 and BT-16 [21]. Both cell lines evaluated showed high sensibility to the virus. Furthermore, the intracraneal administration of rRp450 prolonged the overall survival of an orthotopic AT/RT xenograft model when compared virus treated animals with the vehicle [21].

#### 2.2.4. Vesicular Stomatitis Virus

Vesicular stomatitis virus (VSV) is an enveloped RNA virus belonging to the Rhabdoviridae family that is not known to cause any disease in humans [73]. The wild type VSV exhibit a natural specificity for the replication in cancer cells due to the impairment of the innate immune response in these cells that normally clear the virus after the infection [73,74]. In order to improve the safety of VSV, genetically engineered VSVs were developed and evaluated in several type of cancers [74].

In this sense, Wu et al. assessed the efficacy of the attenuated VSV strain, VSV∆M51 in rhabdoid tumors including AT/RT [35]. VSV∆M51 was generated by the deletion of a single aminoacid of methionine-51 (M51) of the viral M protein increasing the targeting of the virus to tumor cells with defective IFN responses [73]. Human AT/RT cell lines showed an important sensibility to VSV∆M51 correlated with a significant decreased in cell viability after 72 h of infection [28]. Moreover, the treatment with VSVΔM51 decreased tumor size and prolonged survival of CD-1 nude mice bearing BT-16 tumor brain xenografts [35]. In this experiment, the administration of the virus was systemic using the tail vein due to the lethal effect of the virus after intracraneal administration [75]. In this sense, all mice with AT/RT treated with VSVΔM51 eventually died in less than 30 days which suggest that further investigations are needed to improve the efficacy and the safety of VSV for PBTs.

#### 2.2.5. Myxoma Virus

The oncolytic efficacy of myxoma virus has also been studied in AT/RT by Wu et al. showing better results than those obtained with the VSV∆M51 [35]. Both AT/RT cell lines, BT-12 and BT-16, were permissive to myxoma virus infection [35]. Furthermore, a cytopathic effect was evident after 72 h of infection producing an extensive cell death measured by MTT assay [35]. Moreover, in vivo experiments, showed that mice bearing orthotopically BT-16 cell line and treated with an intratumoral injection of myxoma virus showed a significant shrinkage of the tumor compared to control group and that led to a longer survival [35]. In fact, four of six mice treated with myxoma virus were long-term survivors and apparently cured [35].

### 2.3. Gliomas

Glioma is the most common type of brain tumor in adult and children. Low-grade gliomas (LGG) represent the majority of these tumors in children. High grade gliomas (HGG) comprise around 10% of all primary CNS tumors and have the worst prognosis with 7% of 3-year-free survival in children with glioblastoma multiforme [76,77,78].

Despite the recent molecular studies elucidating the genomic make up of pediatric HGGs, the life expectancy of these patients has not improved [79]. Due to the safety profile and efficacy observed in adult gliomas, OVs is a good alternative as a potential therapeutic approach to treat pediatric HGG. In this sense, different types of these viruses have been evaluated in diverse pediatric glioma models.

#### 2.3.1. Seneca Valley Virus

Due to the strong antitumoral effect of SVV-001 previously observed in animal models of MB, the same group evaluated the efficacy of this OV against pHGG [32,33]. To this end, they developed orthotopic xenograft mouse models through direct injection of fresh surgical samples from six pediatric malignant gliomas (five glioblastomas and one anaplastic astrocytoma) into the brains of Rag2/Severe Combined Immunodeficient (SCID) mice [33]. SVV-001 was able to infect, replicate and kill primary cultures of the tumors, neurospheres and self-renewing glioma cells from four mouse models of pediatric gliomas, three glioblastomas and one anaplastic astrocytoma in vitro [33]. Moreover, the i.v. administration of a single dose of SVV-001 in vivo, resulted in a significant increase of the survival time in three orthotopic pediatric glioma xenografts [33].

#### 2.3.2. Parvovirus H-1

The OV, H-1PV has been previously evaluated in MB cell lines in vitro showing an important cytopathic effect [24]. Josupeit et al. also performed in vitro experiments to address the potential of H-1PV as treatment for pHGG [25]. The study determined that the H-1PV efficiently replicates in HGG initiating cells from pediatric glioblastoma and diffuse intrinsic pontine glioma (DIPGs) inducing cytotoxicity [25]. They also showed that H-1PV was able to target both cell subpopulations in the neurosphere cultures, the stem cells and the differentiated cells [25]. This work suggests that H-1PV could be a good therapeutic agent for pediatric gliomas; however, further in vivo experiments are needed to evaluate the safety and efficacy of this OV.

#### 2.3.3. Newcastle Disease Virus

NDV is an enveloped virus with a single-stranded RNA of negative polarity from the paramyxovirus family which naturally infects poultry, with different levels of pathogenicity depending on the virulence of the virus [80]. In humans, NDV is generally non-pathogenic but it can produce minor symptoms including conjunctivitis and flu-like symptoms. Several strains of NDV have shown oncolytic activity in vitro and in vivo in a wide variety of cancers including melanoma, lung and mamarian cancers [10,80,81,82,83,84]. The oncolytic mechanism of NDV has been widely studied and is thought to be dependent on defective interferon responsiveness or cell resistance to apoptosis [11]. In 2004, Csatary et al. reported the administration of the strain MTH-68/H in adult and pediatric patients with recurrent glioblastoma multiforme after the failure of conventional therapies [36]. The report describes the treatment of three children and one adult treated with MTH-68/H that resulted in a significant regression of the tumors and a survival rate of 5–9 years which represents an important increased of the expected survival time for the glioblastoma [36]. A few years later, Wagner et al. also described the case of a 12-year-old child diagnosed with anaplastic astrocytoma WHO Grade III and treated with MTH-68/H strain in combination with valproic acid [37]. The treatment with the OV resulted in an important regression of the tumor, but four months later, a new tumor appeared [37]. Despite the continuous administration of MTH-68/H and valproic acid, the second tumor progressed leading to a fatal outcome [37].

All this clinical data supports the use of NDV as a therapeutic alternative for pediatric gliomas. However, further phase II and III clinical studies with an increasing number of patients would be needed to determine the efficacy of this virus as a real therapeutic alternative for gliomas.

#### 2.3.4. Herpes Simplex Virus

Oncolytic HSV strains have shown to efficiently infect, replicate and lyse diverse types of pediatric tumors including MB and neuroblastoma [17,19,21,85]. Moreover, Ring et al. recently reported that patient-derived pediatric high-grade tumors, including two glioblastomas and one ependymoma xenografts, were more sensitive to G207 than adult glioblastoma xenografts. Furthermore, in vitro and in vivo assays showed that HSV1716 not only produced an antitumoral effect due to the oncolytic effect and the stimulation of the antitumor immune response but also was able to inhibit the migration and invasion of pediatric HGG and DIPG cells used for these experiments [48].

All this preclinical evidence showing the sensibility of PBTs to oHSV and the safety profile and efficacy shown by the HSV G207 strain in the adult clinical trials allowed the design of a phase I clinical trial to evaluate the intratumoral administration of oHSV G207 alone or in combination with a single dose of radiation in children with recurrent or progressive malignant supratentorial brain tumors [86]. This is the first clinical study in human children testing the tolerability and efficacy of the intratumoral inoculation of an OV via catheters placed directly into recurrent or progressive supratentorial tumors [86].

#### 2.3.5. Adenovirus

Ad has been widely evaluated in gliomas. In this sense, the Ad Delta24, has demonstrated a high cytotoxicity in vitro in adult glioma cell lines at 10 MOI within 14 days after infection and an important antitumor effect in glioma models in vivo [60]. Ad infection efficiency is dependent on the expression of CAR in the target cells. In this sense, several tumor cell lines including glioma cells have shown low CAR expression compared to normal cells [87]. In order to enhance the oncolytic activity of Delta24 in glioma, an Arg-Gly-Asp (RGD) motif was inserted into the fiber knob (Delta24RGD) allowing the virus to infect more efficiently through the binding to αvβ3 or αvβ5 integrins which are upregulated in cancer [88,89]. Preclinical studies have demonstrated that Delta24RGD induced a stronger oncolytic effect than the parental Delta24 in primary glioma cells and prolonged the survival of glioma xenograft mice in 60% of animals compared with 15% of Delta24-treated mice [89,90]. Furthermore, the results of a phase I clinical trial to evaluate the safety and efficacy of Delta24RGD (DNX-2401 in the clinic) in recurrent malignant glioma showed that 20% of patients survived more than 3 years without progression from the time of treatment [91]. Moreover, our lab has shown that Delta-24-RGD infects and replicates efficiently in DIPG cell lines inducing a potent cytotoxic effect (IC_50_ ranging from 5 to 50 MOIs) [84] and manuscript in preparation]. All these results constituted the rationale to open a phase I clinical trial to determine the safety and preliminary efficacy of Delta24RGD in pediatric DIPG, which is currently recruiting [31].

## 3. Limitations of Oncolytic Viruses

The studies testing OVs have shown encouraging results and several of these viruses have been evaluated in clinical trials for different types of tumors. However, the use of OVs needs to overcome some important limitations. The most important challenge is the presence of high titers of neutralizing antibodies in the serum of patients against some of the viruses limiting the intravenous administration and, therefore, the use of these viruses in metastasis cancer. In humans, the pre-existing immunity to Ad5 has been reported in around 50% in the United States and Europe, 70% in China and near of 98% in Africa [92,93]. Several reports have demonstrated that the presence of neutralizing antibodies negatively affect the immunogenicity of adenovirus vectors in mice, rhesus monkeys and humans [93]. Moreover, significant levels of neutralizing antibodies have been also detected against HSV, MV and reovirus [94,95]. Nevertheless, the administration of MVs in two seronegative patients showed high titers of neutralizing antibodies 6 weeks after viral administration [96]. In the same line, Evgin, L. et al. observed that the inactivation of vaccinia virus by neutralizing antibodies, after intravenous administration, was dependent on the activation of the complement system [97].

## 4. Future Perspective

Taking together all these promising data supporting the efficacy of OVs for the treatment of different PBTs, clinical trials that evaluate the feasibility and efficacy of these therapeutic candidates in pediatric patients is warranted.

One of the main advantages of OVs over traditional therapies is the few serious adverse effects related to the administration of the viruses in mice and adult patients. In children, the intratumoral administration of Delta-24-RGD (DNX-2401) has been shown to be safe in a case report of an 8-year-old patient with DIPG enrolled in a phase I clinical trial (NTC03178032) to evaluate the safety and the clinical response to the virus [30]. Moreover, in younger patients with recurrent or refractory incurable non-CNS solid tumors the intratumoral administration of the oHSV 1716 was safe and no dose-limiting toxicity was observed (NCT02031965) [98].

All experimental data described in this review have evaluated the efficacy of the OVs alone or in combination with radiotherapy. However, the potential combination with other agents with a complementary mechanism of action that could potentiate the antitumor effect of the different viruses would be extremely interesting [79]. In adults, the combination of OVs with others therapeutic approaches including immune checkpoint inhibitors, CAR-T cells and chemotherapeutic agents have shown promising results in many types of cancer [48,99,100].

On the other hand, an important limitation of the preclinical studies described in PBTs is that all of them have been performed in immunocompromised mice lacking the antivirus immune response which could compromise therapy success. Moreover, in recent years, it became clear that OVs could also induce an important immune response against the tumor which is a factor for the effective response to therapy (Figure 1) [101,102]. In this sense, the development of an antitumoral immune response was important for the response of adult patients with recurrent malignant glioma following a single intratumoral injection of DNX-2401 (Delta-24-RGD) [91]. The results published by Lang F. et al. showed that 20% of patients responded to the therapy. The analyses of the resected tumors revealed an increase in the CD8(+) and T-bet(+) suggestive of a TH1 response [91].

**Figure 2:**
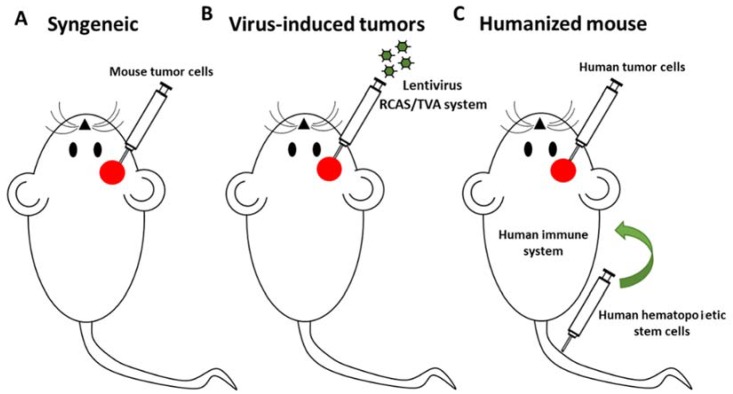
Immunocompetent mouse models for the study of oncolytic virus (OVs) in PBTs. Since the importance of the immune response in the effectiveness of OVs, studies in immunocompetent animal models are needed. Three immunocompetent mouse models are in development to evaluate anticancer therapies, including OVs. (**A**) The generation of mouse tumor cell lines to performed syngeneic immunocompetent mouse models. (**B**) Transduction of specific mouse cell populations with mutated genes to induce tumorigenesis. (**C**) Immunodeficient mice with humanized immune system xenografted with human tumor cells.

In order to improve the antitumor immunity and overcome the antiviral immune response, many OVs armed with immunostimulatory molecules including cytokines (GM-CSF, IL-12, IFNγ), co-stimulatory ligands (OX40L, 4-1BBL), checkpoint inhibitors (anti-PD-1, anti-CTLA4) and even the combination of two molecules have been evaluated with promising results [103,104,105]. The major challenge to evaluate the efficacy and safety of this new generation of OVs in many types of tumors, including PBTs, is the limited immunocompetent models available for the experimentation. In this sense, a big effort has been done to generate immunocompetent transgenic mouse models of different cancers (Figure 2). The classic approach is the use of the syngeneic mouse models which consist in the engraftement of a murine cancer cell line into the same inbred immunocompetent mouse strain (Figure 2A). The main limitation is the lack of murine cancer cell lines representative of the tumors observed in humans and specifically PBTs. One of the novel approaches recently reported is the induction of spontaneous tumors using viral vectors to introduce specific mutations characteristic of human cancers (Figure 2B). Misuraca et al. used the RCAS/Tv-a system to develop a mouse model of pediatric brain stem glioma with the characteristic mutation H3.3K27M to investigate the molecular basis of this disease and novel therapeutic strategies [106]. Moreover, in order to study immunomodulatory therapies in adult gliomas, Marumoto et al. developed an immunocompetent mouse model of glioblastoma by injecting Cre-loxP–controlled lentiviral vectors expressing mutated Harvey Ras and AKT proteins [107]. Humanized mice models engrafted with human hematopoietic stem cells that develop into functional human immune systems have also became an important pre-clinical tool with potential uses in the evaluation of OVs in PBTs (Figure 2C) [108]. All these experimental approaches open exciting possibilities for the pre-clinical evaluation of many OVs required for the subsequent translation to clinic in order to overcome many fatal pediatric cancers.

## 5. Conclusions

The traditional therapies for PBTs are often insufficient for the treatment of many children that nowadays still succumb to these dreadful tumors. Moreover, the survivors develop important sequelae due to the aggressive treatment needed to overcome the disease. In this sense, OVs have demonstrated to be safe and effective therapeutic alternatives supported by numerous pre-clinical and clinical data. Nevertheless, there is still paramount work left to do in order to analyze and improve the immune response induced by OVs against the tumor, a feature that has shown to be a factor affecting the clinical response. Strategies that have been explored in adult brain tumors and other cancer models including novel combinations with checkpoint inhibitors or arming the potential virus with molecules with immunomodulatory and antitumor activity could be tailored to the intrinsic characteristic of the PBTs to achieve maximal efficacy while maintaining a safe profile. The implementation of OVs for the treatment of PBTs could bring hope to these patients.

## Figures and Tables

**Table 1:** Oncolytic viruses evaluated in pediatric brain tumors.

Oncolytic Virus	Name	Genetic Modification	Pediatric Brain Tumor	Development Phase	References
Herpes simplex virus-1	HSV-1716	759 nucleotide deletion in both copies of *RL-1* genes	Medulloblastoma DIPG	Clinical trial (NCT02031965)	[17,18]
	G207	Deletion of both copies of the *g134.5* gene and a lacZ insertion into the *UL39* gene which encodes viral ribonucle- otide reductase (ICP6)	Medulloblastoma Ependimoma Glioblastoma	Clinical trials (NCT02457845)	[18,19,20]
	M002	Deletion of both copies of g134.5 and expresses murine IL-12 constitutively under the transcriptional control of the murine early growth response 1 promoter	Medulloblastoma	Preclinical phase	[19]
	rRp450	Deficient in the viral-encoded ribonucleotide reductase (ICP6)	Medulloblastoma AT/RT	Preclinical phase	[21]
Measles	Edmonston’s strain	Naturally oncolytic	Medulloblastoma AT/RT	Preclinical phase	[20,22,23]
Parvovirus H-1	H-1PV	Naturally oncolytic	Medulloblastoma High grade gliomas DIPG	Preclinical phase	[24,25]
Reovirus serotype 3	Reovirus	Naturally oncolytic	Medulloblastoma	Preclinical phase	[26]
Myxoma	-	Naturally oncolytic	Medulloblastoma	Preclinical phase	[27]
Adenovirus	Delta24	24-base pair deletion in the Rb-binding region of the *E1A* gene	Medulloblastoma	Preclinical phase	[28]
	Delta24RGD (DNX-2401)	24-base pair deletion in the Rb-binding region of the *E1A* gene. Insertion of RGD motif in the fiber knob.	DIPG	Clinical trial (NCT03178032)	[29,30,31]
Seneca Valley-001	NTX-010	Naturally oncolytic	Medulloblastoma Glioblastoma Anaplastic astrocitoma	Preclinical phase	[32,33]
Vaccinia	vvDD	Deletion of thymidine kinase and vaccinia growth factor genes	AT/RT	Preclinical phase	[34]
Vesicular Stomatitis	VSVΔM51	Deletion of methionine 51 in the M protein	AT/RT	Preclinical phase	[35]
Myxoma	-	Naturally oncolytic	AT/RT	Preclinical phase	[35]
Newcastle disease	MTH-68/H	-	Glioblastoma Anaplastic astrocitoma	Preclinical phase	[36,37]
